# Association Between Mean Platelet Volume and Other Inflammatory Markers in Patients with Influenza A (H1N1)

**DOI:** 10.5152/eurasianjmed.2025.25972

**Published:** 2025-12-01

**Authors:** Zeynep Tüzün, Tayyibe Saler, Kemal Tüzün, Ümit Çelik, Begüm Şeyda Avcı

**Affiliations:** 1 Department of Rheumatology, Adana City Training and Research Hospital, Adana, Türkiye; 2 Department of Internal Medicine, Adana City Training and Research Hospital, Adana, Türkiye; 3 Department of Otolaryngology, Seyhan State Hospital, Adana, Türkiye; 4 Department of Pediatric Infectious Diseases, Adana City Training and Research Hospital, Adana, Türkiye

**Keywords:** C-reactive protein, influenza virus, mean platelet volume

## Abstract

**Background:**

Influenza is a viral infection affecting all age groups, with high transmissibility and the potential to cause epidemics and pandemics. Accurate and early prognosis prediction is essential for effective disease management and outbreak control. The objective of the present research was to assess the significance of mean platelet volume (MPV) and its relationship with inflammatory markers, particularly C-reactive protein (CRP), in predicting the prognosis of Influenza A (H1N1) infection.

**Methods:**

A retrospective analysis was conducted on 133 hospitalized patients who presented with respiratory symptoms and tested positive for H1N1 via nasopharyngeal swab. Data on MPV and CRP levels were collected at admission. The presence of comorbidities and clinical outcomes, including mortality, were also recorded. The relationship between MPV, CRP, and patient outcomes was statistically evaluated.

**Results:**

Comorbid conditions were found in 66.2% of the patients. Among the 31 patients who died (23.3%), 77.4% had comorbidities. Mortality was significantly higher in those with comorbidities (27.3%) than those without (15.5%). Compared with the healthy control group, patients exhibited a markedly lower MPV and higher CRP levels (both *P* < .005). Intensive care unit (ICU) patients had higher CRP levels than non-ICU patients (*P* < .005), but MPV did not differ significantly (*P* = .638). Higher CRP was associated with mortality (*P* < .005), whereas MPV showed no significant association (*P* = .086).

**Conclusion:**

H1N1 infection has the potential to cause severe and fatal outcomes, particularly in elderly patients with comorbidities. Elevated CRP at admission may serve as a valuable prognostic marker. Although MPV may contribute diagnostically, its prognostic utility appears limited compared to CRP.

**Main Points**

- To evaluate the impact of seasonal influenza virus infection, which carries high mortality in certain patient groups, on comorbidities and overall mortality.
- To investigate whether inflammation markers at the time of admission can help identify patients likely to have a fatal disease course.
- To assess the role of mean platelet volume, previously studied in many diseases, in influencing the clinical course of influenza infection.

## Introduction

Influenza is an acute, infectious, viral disease characterized by a wide spectrum of diseases, from asymptomatic infection to various respiratory syndromes that may progress to pneumonia.^1^

The infection is more likely to be severe in children under 5 years of age, older adults, White individuals, nursing home residents, individuals with underlying medical conditions such as heart disease or chronic lung disease, those with a history of smoking, and immunocompromised patients.^2^^,^^3^

Influenza is capable of causing severe pneumonia as a primary illness; however, it may also occur concurrently with, or be followed by, a secondary bacterial infection—most often due to *Staphylococcus aureus* or *Streptococcus pneumoniae*.^1^^-^^3^ Influenza A (H1N1) constitutes a major determinant of seasonal influenza activity, accounting for in excess of 200 000 hospital admissions and an estimated 30 000-50 000 deaths annually.^4^ Epidemic outbreaks typically arise every 1-3 years, whereas pandemics are relatively infrequent.^4^ Prompt and precise diagnosis facilitates better clinical management by enabling the early initiation of antiviral treatment and the application of suitable infection control measures.^5^

Laboratory tests are required for a definitive diagnosis. Cultures can be obtained from nasopharyngeal and throat swabs, bronchial aspiration fluids, and sputum samples. Virus RNA can also be isolated from secretions using molecular methods. Serological tests can be performed on blood samples using methods such as hemagglutination inhibition tests. However, these tests are not available in all centers and do not provide rapid results. Faster diagnostic methods are available to support clinician decision-making. Viral antigens can be found by studying samples from respiratory secretions with immunofluorescence, radioimmunoassay, and enzyme-linked immunosorbent assay (ELISA). With ELISA, diagnosis is possible in under an hour. Virus RNA can also be detected using the polymerase chain reaction (PCR) method.^5^

Complete blood count is one of the most routine laboratory tests performed in patients, as it is easy to perform and inexpensive. Mean platelet volume (MPV) is a commonly utilized indicator of platelet size and function, and it is thought to be associated with the degree of inflammation observed in a range of diseases.^6^ When platelets are activated, MPV increases and platelets release inflammatory factors such as chemokines. Furthermore, MPV has been associated with a wide variety of different medical and non-medical conditions.^7^^-^^11^

This study aimed to investigate the effectiveness of MPV and C-reactive protein (CRP) measurements, which can be easily, widely, and quickly applied, in predicting the prognosis of respiratory tract infections caused by the H1N1 virus.

## Materials and Methods

### Study Design

During the seasonal influenza season, the medical records of 800 patients who presented to XXX Hospital with suspected H1N1 virus infection were reviewed. Exclusion from the study was applied to patients who fulfilled any of the following criteria:

- Diagnosed with Influenza B or Influenza A subtypes other than H1N1,
- History of chronic inflammatory conditions, such as rheumatoid arthritis or systemic lupus erythematosus.
- Other active inflammatory or infectious diseases at the time of admission,
- Known hematological disorders or conditions affecting platelet count and function (e.g., thrombocytopenia, myeloproliferative disorders),
- Recent use of medications known to influence platelet indices (e.g., corticosteroids, anticoagulants).

Following these exclusion criteria, 133 patients who were confirmed positive for H1N1 infection via real-time PCR (RT-PCR) assay were enrolled in the study. The control group consisted of 133 healthy individuals matched for age and sex, with no recent infection or inflammatory conditions. The patient and control groups were compared in terms of the CRP and MPV values. In addition, the correlation between the CRP and MPV values and length of hospital stay/disease prognosis was evaluated. All diagnostic and therapeutic procedures were conducted in compliance with the principles outlined in the 1964 Declaration of Helsinki and its subsequent amendments or equivalent ethical guidelines. The study protocol received approval from the Clinical Research Ethics Committee of Adana Numune Training and Research Hospital (Approval no: ANEAH.EK.2016/16, Date: January 27, 2016). Written consent was obtained from the individuals included in the study.

### Diagnostic Analyses

The nasopharyngeal swab samples were sent to the provincial public health laboratory labeled with barcodes containing patient IDs in accordance with the cold chain rules and placed in viral transport medium. Samples that met the acceptance criteria were stored at 4°C and analyzed within 2 days. The RT-PCR testing was used to detect the H1N1 virus after viral RNA was extracted from the swab samples.

The blood sample tubes contained ethylenediaminetetraacetic acid and the MPV values were analyzed using Sysmex XE 2100 analyzers (Sysmex Corp., Hyogo Prefecture, Japan). The measurements were performed 30 minutes after venipuncture. The CRP levels were analyzed using the ROCHE Integra 400 (Roche Diagnostics K.K., Minato‑ku, Tokyo, Japan) with immunoturbidimetric methods.

### Statistical Analyses

Statistical analyses were performed using the MedCalc 16.8.4 (MedCalc Software Ltd., Flanders, Belgium) software package. The normality of the distribution was assessed using the Mann–Whitney *U*-test and the Kruskal–Wallis test. Pearson’s chi-squared test was used to compare demographic data and frequencies between groups. The independent groups *t*-test or Mann-Whitney *U*-test were used according to whether the continuous variables of the 2 groups showed normal distribution or not. Correlation analysis was used to show whether there was a correlation between 2 independent groups. For variables that did not follow a normal distribution, Spearman’s rank correlation coefficient was employed. Statistical significance was defined as *P* < .05.

## Results

Of the patients, 62 (46.6%) were male and 71 (53.4%) were female, including 8 who were pregnant (6.0%). While no additional disease was detected in 45 (33.8%) of the patients, comorbidities were present in 88 (66.2%) (Table 1). Participant ages ranged from 40 days to 87 years, with a median of 31 years. The time between symptom onset and hospitalization was 3.6 ± 2.5 days.

Of the hospitalized patients, 36 required follow-up and treatment in the intensive care unit (ICU) (27.1%). The mean age of the patients admitted to the ICU 44.25 years. The ICU admission indications were as follows: 22 with dyspnea accompanied by hypoxemia, 3 with acute changes in consciousness, 3 with worsening of existing cancer, 1 with diabetic ketoacidosis, 2 with decompensated heart failure, 2 with uremia, 1 with acute stroke, 1 with hyperglycemic nonketotic coma, and 1 with hemolytic anemia.

A mechanical ventilator was required for 21 patients (15.8%). Considering the variability in ventilation duration, the median duration was 2 days. Hemodialysis was applied to 9 patients (6.8%). The median length of stay for the patients hospitalized in the ICU was 5 days. Moreover, 94 (70.7%) of the patients were discharged after recovery, 31 (23.3%) died, 6 (4.3%) were discharged voluntarily, and 2 (1.5%) were referred. Furthermore, 31 had a fatal outcome, and among these, 24 had comorbidities. The mortality rate was 27.3% in patients with comorbidities and 15.5% in those without.

The mean age of the patients with a fatal outcome was 50.48 ± 26.17 years. The comorbidities of the patients who died included 7 with active cancer, 4 with diabetes (including 1 with diabetic ketoacidosis), 2 with chronic kidney failure, 2 with acute kidney failure, 1 pregnancy, 1 asthma, 2 with metabolic syndrome, 2 with coronary artery disease, 2 with heart failure, and 1 with hemolytic anemia. While 11 (30.5%) of the 36 patients hospitalized in the ICU were discharged after recovering, 25 (69.5%) died. Moreover, 18 (72%) of the 25 patients who died in the ICU had an additional disease.

The mean duration of antiviral treatment was 5.3 ± 2.6 days. Antiviral treatment was continued until 2 days after the patient’s complaints were resolved. Supportive treatment and treatment of comorbidities were given to the patients. In addition to antiviral treatment, patients with worsening respiratory symptoms were treated with steroids equivalent to 40 mg/day methylprednisolone and supportive treatments.

No significant changes were observed in the counts of white blood cells, including neutrophils, monocytes, and lymphocytes. In contrast, eosinophil counts showed a significant increase following treatment (Table 2).

**Figure 1.**
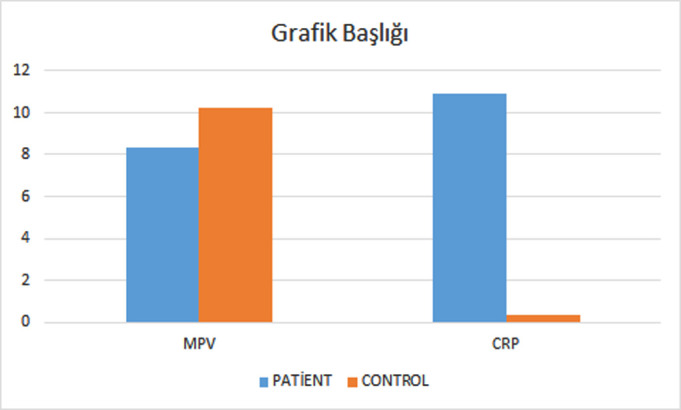
Comparison of the MPV and CRP values in the control and patient groups.

When the MPV and CRP values of the patient and control groups were compared, 5 (3.7%) of the patients were not included in the comparison since the MPV could not be read due to significant thrombocytopenia. When compared to the control group, the MPV was lower in the patient group, while the CRP level was higher (*P* < .005 for both) (Figure 1).

**Figure 2.**
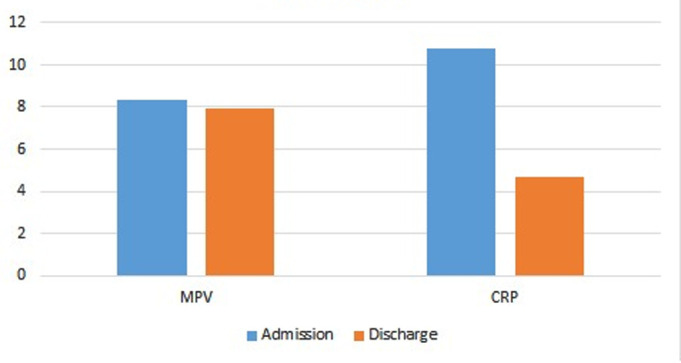
MPV and CRP values during hospitalization and at discharge.

The MPV and CRP values measured at hospital admission were compared with those measured at discharge, and both were significantly decreased at discharge (*P* < .005 for both) (Figure 2). A comparison of MPV and CRP values between ICU and inpatient service patients demonstrated a statistically significant elevation in CRP levels in the ICU group (*P* < .005), whereas the MPV values showed no significant variation between the groups.

**Figure 3.**
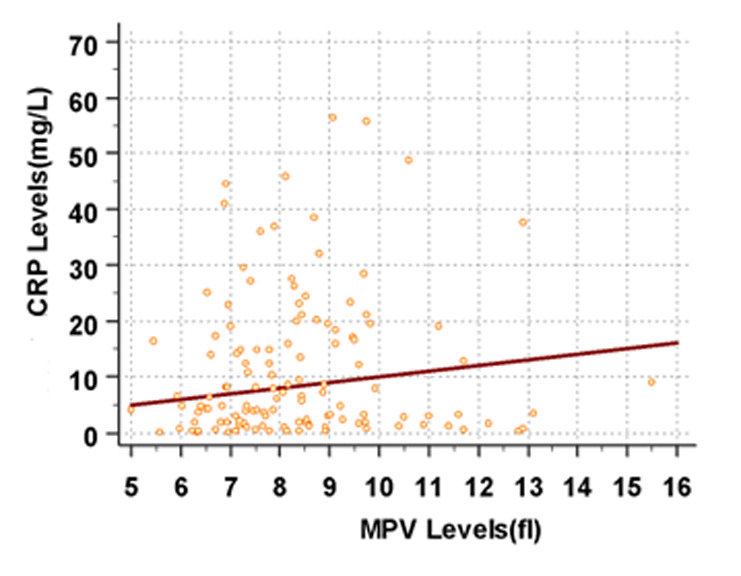
Correlation between the MPV and CRP values at the time of hospital admission.

No significant correlation was observed between MPV and either the length of hospital stay or the requirement for mechanical ventilation. Similarly, no correlation was found between the CRP levels and the length of hospital stay or the need for a mechanical ventilator. The CRP levels at admission were significantly associated with mortality (*P* < .005), whereas no statistically significant correlation was found between admission MPV and mortality (*P* = .086). On the contrary, the decrease in MPV demonstrated a very weak negative correlation with the increase in CRP levels (correlation coefficient: *r* < 0.06) (Figure 3).

## Discussion

The diagnosis of influenza may be difficult in mild to moderate cases, as such presentations are clinically indistinguishable from influenza-like illnesses caused by other respiratory viruses. Moreover, the lack of reliable parameters to predict diagnosis and prognosis remains a major challenge for H1N1 infections.^12^^,^^13^ The relationships between CRP and MPV values in H1N1 infection have not yet been fully investigated.

In line with existing literature, the current study found that ICU admission and mortality rates were higher in patients of advanced age and those with comorbidities. This can be explained by the decline in immune defenses and the increased prevalence of accompanying diseases with aging. Influenza can cause primary viral or secondary bacterial pneumonia and, in healthy older adults, lead to serious cardiovascular events such as myocardial infarction or neurological complications like stroke. Additionally, influenza may worsen underlying chronic conditions such as congestive heart failure, chronic obstructive pulmonary disease, asthma, and diabetes.^14^

The analysis revealed that MPV in the patient group was statistically significantly lower compared with the control group. Furthermore, a very weak inverse correlation was identified between the decrease in MPV and increase in CRP levels. This finding aligns with previous reports suggesting that while the MPV may reflect platelet activation and inflammatory status, its relationship with systemic inflammation markers such as CRP is complex and may not be robust. The weak correlation observed might be attributable to the multifactorial regulation of MPV during acute viral infections, where platelet size and reactivity can be influenced by a variety of host and pathogen factors. Moreover, CRP, as an acute-phase reactant, more directly reflects the systemic inflammatory response, potentially explaining its stronger association with disease severity and outcomes in H1N1 infection. These observations underscore the limitations of MPV as a standalone prognostic marker and highlight the need for further studies employing serial MPV measurements and standardized methodologies to better elucidate its clinical utility in influenza and other viral infections.^11^^-^^15^ A similar study by Yüksel et al^6^ investigated the MPV levels in patients with inflammatory bowel disease. They found that the MPV levels were significantly lower in patients with active disease compared to those in remission and suggested that the MPV could serve as an inflammatory marker reflecting disease activity and severity. In the current study, both CRP and MPV showed potential as diagnostic tools in H1N1 infection. With further refinement and standardization, potentially via machine learning algorithms, these biomarkers could contribute to earlier and more accurate diagnosis and treatment.

However, no significant difference in MPV was found between survivors and non-survivors, whereas CRP levels were significantly elevated in patients who died. Akarsu et al^16^ previously found lower MPV levels in neonates who died from sepsis, indicating its potential prognostic value in certain settings. The lack of prognostic significance of MPV in the study may be explained by differences in timing between symptom onset and hospitalization, individual variability, and the limitations in the MPV’s reliability as a marker. Additionally, the relatively narrow dynamic range of MPV and the lack of serial MPV measurements may have limited its utility. Future studies that include repeated early-phase MPV measurements may better elucidate its prognostic value. Moreover, the study included a wide age range, and hematological parameters may vary significantly across age groups. This variability should be considered a limitation of the study. On the other hand, the study included a relatively large and diverse patient population, strengthening the generalizability of the findings. These advantages are highlighted in the Discussion section.

When the MPV at admission and discharge were compared, a significant decrease was observed at discharge. This contrasts with older studies from the 1990s, which reported increased MPV at discharge likely due to methodological differences. A meta-analysis of 11 studies revealed significant variability in MPV results due to differences in measurement techniques, anticoagulants used, and time elapsed between venipuncture and analysis.^17^^,^^18^ As platelets swell and become rounded within approximately 1 hour after sampling, MPV readings may be affected. Therefore, discrepancies in sample handling, comorbid conditions, disease severity, and measurement timing may all influence MPV values.^17^^-^^19^

H1N1 infection can be severe and fatal, particularly in elderly patients and those with underlying health conditions. However, severe illness can also occur in individuals without known risk factors, highlighting the importance of early prognostic indicators. The findings suggest that CRP levels at admission may serve as a reliable prognostic marker and help guide decisions regarding hospitalization and treatment. While MPV has been associated with inflammatory and malignant diseases in prior literature, its role in determining disease severity in H1N1 appears to be limited compared to CRP. Nevertheless, MPV may still serve as a supportive diagnostic marker, though its clinical utility requires further validation through standardized and repeated measurement protocols.

## Figures and Tables

**Table 1.** Demographic Characteristics of the Patients: Comorbidities

Additional Disease	Patients (n)	Percentage (%)
Without comorbidities	45	33.8
Chronic renal failure	7	5.3
Diabetes mellitus	9	6.8
Active cancer	14	10.5
Pregnancy	8	6.0
Congestive heart failure	3	2.3
Coronary artery disease	2	1.5
Acute coronary syndrome	3	2.3
Chronic obstructive pulmonary disease	3	2.3
Metabolic syndrome	3	2.3
Acute renal failure	4	3.0
Hemolytic anemia	6	4.5
Stroke	1	0.8
Asthma	4	3.0
Mental motor retardation	4	3.0
Other disease	17	12.6

**Table 2.** Comparison of the Laboratory Parameters Before and After Treatment

Laboratory Parameter	Pretreatment (Mean ± SD)	Posttreatment (Mean ± SD)	*P*
White blood cells (×10^3^/µL)	9.5 ± 6.2	9.9 ± 6.6	.876
Monocytes (×10^3^/µL)	0.75 ± 0.95	0.56 ± 0.38	.942
Neutrophil (×10^3^/µL)	6.7 ± 5.4	6.8 ± 6.7	.406
Lymphocyte (×10^3^/µL)	1.99 ± 1.97	2.18 ± 1.96	.053
Eosinophil (×10^3^/µL)	0.04 ± 0.007	0.20 ± 0.68	<.005
Platelet (×10^3^/µL)	229.4 ± 128.7	287.9 ± 159.2	<.005
MPV (fL)	8.3 ± 1.7	7.9 ± 2.0	<.005
Albumin (g/dL)	3.6 ± 0.7	3.2 ± 0.7	<.005
CRP (mg/L)	10.8 ± 12.7	4.7 ± 9.1	<.005

## Data Availability

The data that support the findings of this study are available on request from the corresponding author.
